# Mechanical assessment of novel compliant mechanisms for underactuated prosthetic hands

**DOI:** 10.3389/fbioe.2023.985901

**Published:** 2023-10-11

**Authors:** Orion Ramos, Laura de Arco, Carlos A. Cifuentes, Mehran Moazen, Helge Wurdemann, Marcela Múnera

**Affiliations:** ^1^ School of Engineering, Science and Technology, Universidad Del Rosario, Bogota, Colombia; ^2^ Telecommunications Laboratory (LABTEL), Electrical Engineering, Federal University of Espirito Santo (UFES), Vitória, Brazil; ^3^ Bristol Robotics Laboratory, University of the West of England, Bristol, United Kingdom; ^4^ Department of Mechanical Engineering, University College London, London, United Kingdom; ^5^ Biomedical Engineering Department, Colombian School of Engineering Julio Garavito, Bogota, Colombia

**Keywords:** compliant mechanism, hand prosthetics, soft-actuators, mechanical assessment, grasping

## Abstract

This paper proposes novel compliant mechanisms for constructing hand prostheses based on soft robotics. Two models of prosthetic hands are developed in this work. Three mechanical evaluations are performed to determine the suitability of the two designs for carrying out activities of daily living (ADLs). The first test measures the grip force that the prosthesis can generate on objects. The second determines the energy required and dissipated from the prosthesis to operate. The third test identifies the maximum traction force that the prosthesis can support. The tests showed that the PrHand1 prosthesis has a maximum grip force of 23.38 ± 1.5 N, the required energy is 0.76 ± 0.13 J, and the dissipated energy is 0.21 ± 0.17 J. It supports a traction force of 173.31 ± 5.7 N. The PrHand2 prosthesis has a maximum grip force of 36.13 ± 2.3 N, the required energy is 1.28 ± 0.13 J, the dissipated energy is 0.96 ± 0.12 J, and it supports a traction force of 78.48 ± 0 N. In conclusion, the PrHand1 prosthesis has a better performance in terms of energy and tensile force supported. The difference between the energy and traction force results is related to two design features of the PrHand2: fully silicone-coated fingers and a unifying mechanism that requires more force on the tendons to close the prosthesis. The grip force of the PrHand2 prosthesis was more robust than the PrHand1 due to its silicone coating, which allowed for an improved grip.

## 1 Introduction

As the number of amputees and people with upper extremity disabilities continues to grow, robotic hands are increasingly viewed as a solution to improve their quality of life (Jelačić et al., 2020). According to current estimates, it is expected there will be 3.6 million amputees in the United States alone by 2050 (Jelačić et al., 2020). In developing countries, the number of amputees without access to an assistive device was estimated at 30 million (del Carmen Malbrán, 2011). According to Colombia’s 2020 social protection report *SISPRO*, there were more than half a million people with mobility disabilities in their upper limbs (34.93% of the Colombian population) (SISPRO 2020).

Upper limb amputations generate numerous issues, including reduced self-esteem and physical problems that can prevent a person from performing activities of daily living (ADL) (Ziegler-Graham et al., 2008; Daniels et al., 2020). Therefore, the development of devices such as robotic hands aims to help people with disabilities (Robinson et al., 2014). However, a significant portion of the world’s vulnerable population cannot access these types of devices, further deepening health inequality (Borg and Östergren, 2015).

Prosthetic devices replace the missing part of the human hand, and the main objective of prosthetic devices is to achieve the best possible functionality to help people with disabilities, regardless of aesthetics. In addition, prosthetic manufacturers seek to reduce costs and improve manufacturing methods to make the devices more widely accessible (Tian et al., 2017a). In the field of robotics, the construction of assistive hand devices seeks to combine the functionality of the prosthesis with the application of new technologies to achieve similar functioning as the human hand (Huaroto et al., 2020). Robotic hands have evolved from devices with limited functionality to systems capable of understanding and replicating human hand movements (Salvietti, 2018). The improvement of functionalities is related to increasing degrees of freedom (DoF) and different manufacturing technologies (Cordella et al., 2016). Previously, robotic hands were manufactured with rigid elements and industrial materials that generated heavy devices (Shintake et al., 2018). Actuation methods were based on motors and gears, which were required for each DoF of the device (Zappatore et al., 2017). A number of new techniques have emerged to reduce the weight and facilitate the manufacturing process of the devices. These processes include 3D printing technologies, new actuation techniques (Ten Kate et al., 2017), the use of motors to pull joint-actuating tendons (Jing et al., 2019), and design techniques that reduce the number of actuators needed for DoF (i.e., underactuated devices) (Massa et al., 2002; Niola et al., 2014). Finally, the inclusion of new soft materials in the manufacturing process has also reduced the fabrication time, cost, and weight of the devices (Polygerinos et al., 2017).

Robotic hands built using 3D printing can be divided into two classes: those that use pins as joints in rigid parts (Wahit et al., 2020) and those that use compliant mechanisms and flexible materials (Mutlu et al., 2015). Tendon drive techniques can be used in both types of joints, allowing for the reduction of actuators in this type of device. Soft robotics—devices using soft actuators and materials—is another option. For example, the design of fingers with silicone actuators for a robotic hand (Fras and Althoefer, 2018), improvements in grip functionality with specialized actuators for the palm (Li et al., 2019), or others where the type of actuator and its control is evaluated (Deimel and Brock, 2016; Tian et al., 2017b).

The use of techniques based on soft robotics aims to solve significant challenges faced by conventional robotic hands. For example, soft robotics allows for weight reduction, faster production, and improve safety human-device interaction (Laschi et al., 2016). However, the new technology cannot yet fully replace a conventional prosthesis due to the maximum force it is capable of exerting and the complexity of control involved in these actuators (Laschi et al., 2016). Soft materials have infinite DoF, which causes the joints to bend in undesired directions during force execution. However, soft joints with compliant mechanisms and tendon routing can establish fully controlled actuation. Links with guided soft joints can move and exert forces on predefined axes. However, they can flex and adapt to the environment when perturbations are greater than a certain threshold. This property makes manipulators with compliant mechanisms more adaptable and durable (Liu et al., 2018).

Current state-of-the-art prosthetic technologies rarely implement degrees of abduction of the middle fingers, especially in devices with rigid components (Owen et al., 2018), with existing devices using gear and motor methods for this DoF (Vulliez et al., 2018). Robotic hands using compliant mechanisms have also implemented the degree of abduction in the main fingers. In this technology, robotic hands have been developed with passive degrees of abduction, i.e., they are not controllable. Passive DoFs only give a plus in drop tolerance, and increased adaptation in grasping objects (Liu et al., 2019; Mottard et al. 2017). In the case of soft-actuated robotic hands, two types of abduction are observed: passive abduction and abduction joint designed to be actuated. In the former, the soft material itself generates abduction by having infinite DoF. Therefore, the degree of abduction exists but without being designed to be actuated (Godfrey et al., 2018). The other type of abduction is when the joint is designed to be actuated. In most cases, this occurs pneumatically using silicone actuators. A design of this joint can generate different motions that increase the dexterity and functionality of the robotic hand (Tian et al., 2017a; Xu et al., 2018).

The addition of abduction degrees of freedom and the use of new manufacturing ultimately aim to improve users’ quality of life by improving the performance of robotic hands. However, these devices face complex challenges. Devices made of rigid materials must become more compatible with humans, and devices based on soft robotics must be able generate higher forces and better control.

Mechanical tests allow for the evaluation of the mechanical capacities of the prosthesis and its resistance to the different loads encountered as part of ADL. One of the most commonly used tests to determine maximum mechanical capacity is grip force, which measures the force that the prosthesis can generate on a grasped objects (Cuellar et al., 2020). A grip force of 10 N is considered sufficient to carry almost all objects related to ADL (Smit and Plettenburg, 2010a), and most of the current developments manage to exceed this value (Cuellar et al., 2020 Moreo, 2016).

In any electromechanical device, the aim is to reduce the amount of energy required and dissipated from the system. Therefore, if two devices performing the same task are compared, the one requiring less energy is considered more efficient. Similarly, the less energy the system dissipates, the more efficient the mechanisms are. This is because they transfer the input energy better and do not dissipate it in the form of heat or other energy. For example, in tendon-actuated prostheses, the dissipation of energy by transmission should be minimal. This reduces the input effort required by the actuator and produces a higher grasping force. This mechanical test determines the system’s efficiency by finding the energy needed to close the hand and how much of the energy is lost in the operation of the prosthesis (Smit et al. 2015). The energy ranges (both required and dissipated) in current hands are around 1,058–2,292J (Smit and Plettenburg, 2010a).

The traction test is intended to determine the maximum force that the prosthesis can be subjected to. The traction force appears when a vertical force opposite to the force exerted by the hand is generated, e.g., when carrying a market bag or lifting a heavy object. The traction test typically takes the mechanisms to their limit by generating permanent mechanical damage, such as shaft breakage or deformation of prosthesis parts. Based on the Yale-CMU-Berkeley (YCB) standardized object set (Calli et al., 2015), the heaviest object used as part of ADL measurements weighs 3.1 kg, so a minimum traction force of 30 N is required to hold an object of this weight without damage.

These tests allow the mechanical comparison of different prosthesis designs. Therefore, this study aims to determine whether new construction techniques based on soft robotics and compliant mechanisms can build a prosthesis capable of meeting the mechanical requirements of grip force, energy, and traction force for ADL requirements. To investigate this research question, two prostheses with design differences will be constructed and evaluated mechanically.

## 2 Methodology and materials

**FIGURE 1:**
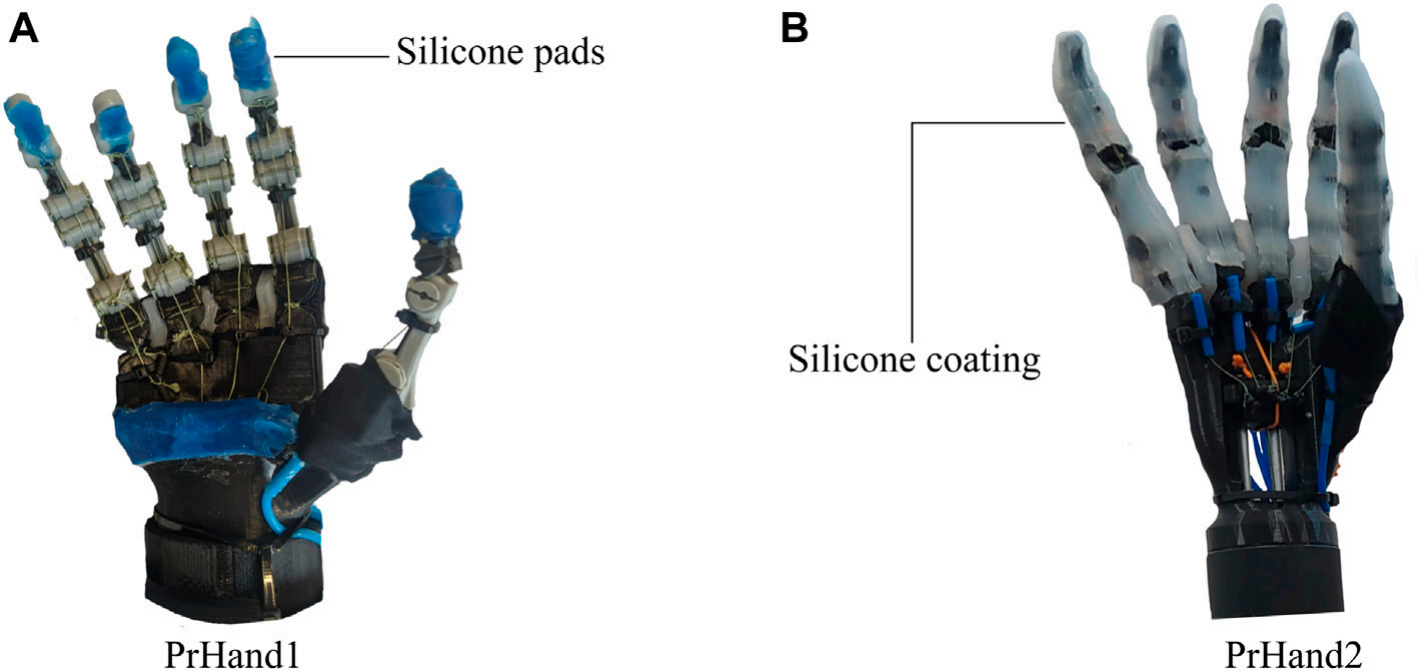
The hand prostheses constructed for this study are based on compliant mechanisms and soft actuators. **(A)** The PrHand1 prosthesis has silicone coatings only at specific points. **(B)** PrHand2 prosthesis has fingers completely coated with silicone.

In this study, two prostheses were constructed using compliant mechanism techniques and soft actuators (see Figure 1). Both designs are constructed with guided soft joints (Culha et al., 2017). Each finger link is made of rigid material (PLA) and 2.85 Filaflex tendons (Recreus, España) with elastic properties to perform their actuation. Finger abduction is achieved by the force generated by pneumatic silicone actuators. The two designs use a Dynamixel MX-106 motor (Robotis, USA) to actuate the flexion of the five fingers and an air pump (MITSUMI, Japan) to pressurize the abduction actuators. The system for the two designs uses a 12 V, 5A supply for operation. The hand control was performed in ROS on a Raspberry Pi 3.

**FIGURE 2:**
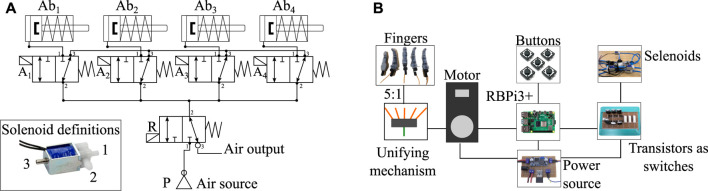
Pneumatic and electronic control systems used in both prostheses. **(A)** Diagram of pneumatic connections to control the soft finger abduction actuators. P symbolizes the air pump, R the air retention solenoid valve, and A(_1⋯4_) are the solenoid valves for each actuator Ab(_1⋯4_). **(B)** Electronic control scheme responsible for manipulating the flexion of the fingers from the motor of the prosthesis and controlling the solenoid valves of the pneumatic actuators through transistors.

Both devices use the same pneumatic and electronic control system since the same components are used for their actuation. To achieve the abduction movements in the prostheses, silicone actuators controlled by open three-way mini-solenoid valves with two positions for each actuator are used (Generic 3/2, China). Each solenoid valve is actuated through the Raspberry Pi independently according to the type of grip to be performed. The general pneumatic connection of the solenoid valves A(_1⋯4_), air pump (P) and actuators Ab(_1⋯4_) is shown in Figure 2A. To reduce energy consumption, the activation of the air pump is also controlled using the Raspberry Pi. The pump is only turned on when pressure is required by the actuators.

As with the pneumatic control system, the two prostheses share the same electromechanical control scheme. The entire system is powered by a 12-volt supply passing through a regulator that provides 5 volts to power the various electronic components. Since only one motor is used in the design of these devices for the flexion of the fingers, the five tendons (one per finger) must be unified into a single tendon tensioned by the motor. This system is called a unifying mechanism, and this mechanism is different in each version of the prosthesis. Both the flexion of the fingers and the activation of the pneumatic actuators are controlled by physical push buttons. The electromechanical scheme shared by the two devices can be seen in Figure 2B.

### 2.1 Novel finger compliant mechanism joint

**FIGURE 3:**
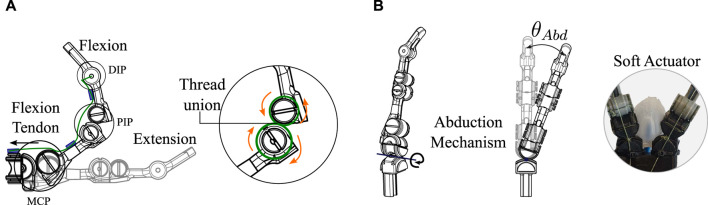
Novel compliant mechanism used for flexion, extension, and abduction of the fingers of the two prostheses. **(A)** Explanation of how the finger flexion and extension are generated based on the compliant mechanism of two tangential circumferences at a single point. **(B)** Abduction degree of freedom driven by a soft silicone actuator.

The main characteristic of the prosthesis design under investigation is the flexion and extension mechanism of the fingers. The mechanism is based on compliant mechanisms constructed from a single material (Hill and Canfield, 2016). However, two circumferences are used, joined by a tension element such as a rigid thread (Sufix 832, USA) that allows rigid materials for its construction, such as PLA. The finger flexion is achieved using the tension force generated to the joint through a tendon, as shown in Figure 3A. Unlike a traditional revolute joint, this joint does not rotate about a fixed axis; instead, the joint rotates and translates tangentially about a circumference. This novel joint is more similar to joints in the human body (Xu and Todorov, 2016). These properties facilitate construction and assembly by making the joint’s exact alignment with an axis unnecessary. The faces of the two circumferences only touch at one point in their movement; there is no friction and, therefore, no wear. Finally, another advantage of this joint is due to the union of the two circumferences being made with thread. Although it is rigid, the joint allows small deformations in situations of shocks or great forces, preventing any part from breaking. Two of these novel joints were used to flex and extend the metacarpophalangeal (MCP) and interphalangeal (PIP) joint of the finger. The distal interphalangeal (DIP) joint in these designs was not constructed to be a degree of freedom. The location of the joints are seen in Figure 3.

This same novel mechanism is used for the degree of freedom of abduction in the fingers of the two prostheses. Unlike the flexion and extension degrees already presented, the degree of abduction is driven by a force generated by the silicone actuators, as shown in Figure 3B. In this case, the tangent circumferences are at 90 degrees to the axis of the flexion and extension joints. The final angle of abduction (*θ*_*Abd*_) of each finger depends on how much pressure is applied to the pneumatic actuators. In this application, the angle *θ*_*Abd*_ does not require any sensor or control to be generated; only the timing of the solenoid valve is defined to allow air to enter the actuator.

**FIGURE 4:**
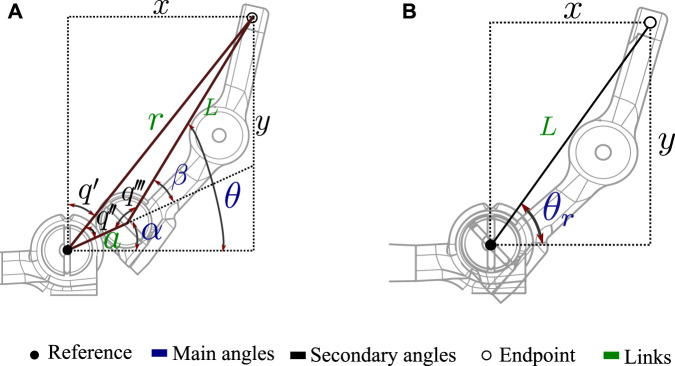
Variables involved in the kinematic solution of articulations. **(A)** Variables of the novel joint based on the compliant mechanism used in the prostheses constructed in this study. **(B)** Related variables in a traditional revolute joint.

By not behaving as a typical revolute joint, different behavior in flexion and extension of the fingers is exhibited. To kinematically explain how flexion is generated with this joint, the forward and inverse kinematics equations are calculated. PIP degree of freedom is used for the explanation as presented in Figure 4A. The reference point of the system and the endpoint (x,y) for the kinematic calculation of the joint can be seen in Figure. The variables required for the joint kinematic solution include principal angles *θ*, *α* and the links’ distances, (*a*, *L* and *r*).

The equations that determine the final coordinate (x, y) of the link to the principal angle *θ* in the novel joint can be seen in Eq. 1. Unlike the solution of a traditional revolute joint (Eq. 2), these equations are composed of the two terms that depend on the angle *θ* and *α*. Where

$$\alpha =\frac{R_{2}}{R_{1}+R_{2}}\theta ,$$

$$a=R_{1}+R_{2}$$

and *R*_1_, *R*_2_ are the radius of the circumferences.

$$\left[\begin{array}{c} x\\ y \end{array}\right]=\left[\begin{array}{c} a\cos\left(\alpha \right)\\ a\sin\left(\alpha \right) \end{array}\right]+\left[\begin{array}{c} L\cos\left(\theta \right)\\ L\sin\left(\theta \right) \end{array}\right]$$ (1)

The variables involved in a typical revolute joint can be seen in Figure 4B. The forward kinematics of this joint is presented in Eq. 2. The equations show how only one term is dependent on *θ*_*r*_ and defines the final coordinate (x, y).

$$\left[\begin{array}{c} x\\ y \end{array}\right]=\left[\begin{array}{c} L\cos\left(\theta_{r}\right)\\ L\sin\left(\theta_{r}\right) \end{array}\right]$$ (2)

To complete the kinematic solution of the proposed joint for prosthetic fingers, Eq. 3 shows the inverse kinematics solution. Where *α* depends on the (x, y) coordinates as seen in Eq. 4 and *β* only depends on known joint parameters (Eq. 5). Moreover, Eq. 6 shows the solution of the inverse kinematics of the revolute joint.

θ=β+α (3)

$$\alpha =90-\arctan\left(\frac{x}{y}\right)-\arccos\left(\frac{r^{2}+a^{2}-L^{2}}{2ar}\right)$$ (4)

$$\beta =180-\arccos\left(\frac{a^{2}+L^{2}-r^{2}}{2aL}\right)$$ (5)

$$\theta_{r}=\arctan\left(\frac{y}{x}\right)$$ (6)

These equations can be used to compare the behavior of the novel joint based on a compliant mechanism and a traditional revolute joint. Moreover, this approach to kinematics allows the entire motion of the fingers to be calculated in series by using only the two flexion degrees of freedom (MCP and PIP).

The kinematic equations are important for understanding the movement and behavior of the joint, which can be beneficial for optimizing the design and performance of the prosthesis in the future. Furthermore, it is important to establish a baseline for the kinematics of this new mechanism in order to have a reference point for future studies and comparisons. Overall, while the kinematic equations may not be necessary for evaluating the immediate performance of the prosthesis, they provide valuable information for understanding and improving the technology in the long term.

The similarities between the designs include the novel compliant mechanism for the degrees of freedom of the fingers, the actuators used, the pneumatic scheme, and the electromechanical control scheme. The key differences between PrHand1 and PrHand2 are: the number of soft actuators used for abduction, the location of the actuators, the actuation of the thumb, the coating of the fingers to increase grip friction, the location of the extension tendon, and the unification system. A detailed description of each prosthesis is presented below.

#### 2.1.1 PrHand1

**FIGURE 5:**
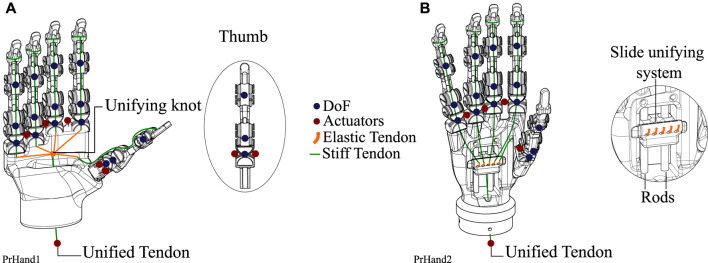
Design differences between the two constructed prostheses. These include the DoF, the number of actuators used, and the unification system of five tendons to one. **(A)** PrHand1 prosthesis description where six control actuators and the tendon unification knot are shown. **(B)** The PrHand2 prosthesis with five control actuators and a sliding mechanism to unify the tendons.

The PrHand1 design uses an Ecoflex 00–50 silicone coating in localized parts of the fingers and palm of the prosthesis, which do not generate any restriction in the flexion movement (see Figure 1A). In this version, five soft actuators are used to control the abduction degrees of freedom. The thumb’s initial position in the PrHand1 prosthesis is 45 degrees to the palm, as is the thumb of the human hand (Huang and Huang, 2019). This initial position requires two soft actuators to control the abduction and adduction of the thumb. Each prosthetic finger of the PrHand1 design has three degrees of freedom: two degrees of freedom of these (PIP and MCP) are for flexion of the prosthetic fingers. The other degree of freedom is for the abduction of the central fingers. This distribution of the joints in the main fingers does not mimic the DIP degree of freedom in the human hand. In this design, this joint is fixed with an angle of 30° degrees. In total, this prosthesis design has 15 degrees of freedom distributed in the fingers only (see Figure 5A).

An elastic tendon pulled by a single motor moves the PIP and MCP joints of the four main fingers simultaneously. Likewise, the tension in the tendon moves the IP and MCP joints of this finger, together with the thumb, to generate flexion. The unifying system of the five tendons connects to a single tendon which is driven by the motor pulley is a knot that collects the tendons, as seen in Figure 5A. To achieve low-level independence in each finger, elastic tendon sections were used, allowing for independent deformation of each finger. When flexion is generated, the fingers take the shape of the object they are holding. The longer the elastic tendon section, the more compliant the grip, and the less force it can exert. The size of the elastic tendon can be modified by simply making the union knot at a different point, thus lengthening or shortening the distance between the elastic tendons.

**FIGURE 6:**
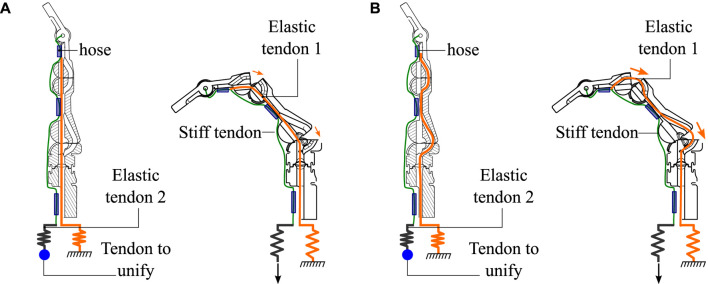
Internal view of the fingers used in the prostheses in the neutral and flexion positions. **(A)** The ducts used by extension tendon 1 in the PrHand1 prosthesis and the forces generated in the flexion movement. **(B)** The ducts used by extension tendon 1 in the PrHand2 prosthesis and the forces generated in the flexion movement.

The fingers of the PrHand1 prosthesis have an internal elastic tendon that generates a counter force to flexion to avoid the need for an actuator responsible for finger extension. This tendon is called the extension tendon. This design is located on the center or neutral line of the flexion motion, as seen in Figure 6A. Thus, the extension tendon does not generate high opposing forces to the flexion motion.

#### 2.1.2 PrHand2

The PrHand2 design uses Ecoflex 00–50 silicone coating around the fingers except for the joints. This is because the silicone generates high flexion restriction (see Figure 1B). In this version, four soft actuators are used only to control the abduction degrees of freedom, and, unlike the PrHand1 prosthesis, the initial position of the thumb is located at 90 degrees to the palm. This was done to remove a soft actuator and reduce the complexity of grip types. Like the PrHand1 design, each prosthetic finger of the design has three degrees of freedom: two degrees of freedom (PIP and MCP) are responsible for the flexion of the prosthetic fingers, and the other degree of freedom is to generate abduction in the main fingers. This distribution of the joints in the main fingers does not take into account the DIP degree of freedom of the human hand. In total, this design has 15 degrees, as seen in Figure 5B.

The unifying mechanism in the PrHand2 prosthesis is more complex than the unifying knot of the PrHand1 design. For this prosthesis, a sliding mechanism was used to collect the elastic tendons of the fingers and attach them to a moving part that slides using two parallel rods. The movement of the mobile piece is generated by the rigid tendon connected to the motor (Figure 5B). As in PrHand1, elastic tendons were used to generate compliant grips in the PrHand2 prosthesis. In this case, the unifying mechanism of this prosthesis holds five elastic tendons, one for each finger. The unifying mechanism then transforms the five elastic tendons into a single rigid tendon pulled by the motor. In this mechanism, the elastic elements are the same size for each finger. However, modifying their size to allow for more or less deformation is not possible after the mechanism is assembled. This system allows the assembly and attachment of each tendon to be independent, which makes the assembly of the device more practical than the PrHand1 prosthesis.

Internally, the fingers of the PrHand2 prosthesis differs from PrHand1 because the extension tendon does not pass through the center or neutral line of the joint. Instead, the extension tendon is displaced to the limit of the motion circumferences, which generates a force that returns the joint to its original position (Figure 6B). This displacement increases the force required by the motor to achieve finger flexion. The overall dimensions of the two prostheses and the number of actuators used in each design can be seen in Table 1.

**TABLE 1:** Summary of mechanical properties of the proposed designs.

Property	PrHand1	PrHand2
Palm length [cm]	10	8.1
Hand length [cm]	19	19.5
Palm width [cm]	8.5	6.8
Palm depth [cm]	4	2.8
DoF	15	15
Actuators	6	5

In this section we delve deeper into the methodology used to evaluate the PrHand1 and PrHand2 prostheses from a mechanical perspective. In particular, the configurations and sensors used to measure the various variables in subsections 2.2, 2.3 and 2.4 are examined. In addition, subsection 2.5 provides a detailed explanation of the statistical analysis methodology used to classify and compare the results of each test.

### 2.2 Grasping force

**FIGURE 7:**
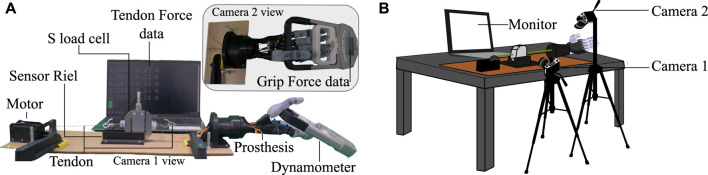
Test bench used for grip force tests and the calculation of energy used for the grip used in this study. **(A)** Description of each component used in the setup; the view of each camera for data processing is shown. **(B)** Location of the cameras used as sensors for the grip force test setup.

This test aims to find the maximum grip force achieved by hand prostheses driven by tendons and motors. Determining the maximum grip force in the constructed prostheses was based on applying the maximum force of the motor and measuring the generated grip force in the fingers of the prosthesis with a hand dynamometer (EH101 CAMRY, USA) (Cuellar et al., 2020; Cuellar et al., 2019). The variables of this experiment are the maximum force generated in the grip and the maximum force generated in the flexion tendon. A standard hand dynamometer (EH101 CAMRY, USA) was used to measure the grip force in kg, and an S-type load cell (50 kg Lexus, China) was used to measure the tension in the flexion tendon of each prosthesis. The acquisition of the load cell data requires an HX711 amplifier (Avia semiconductor, China) which is collected by an Arduino UNO (Arduino, USA). The drive motor must be decoupled from the prosthesis to locate the S-type load cell between the unifying mechanism and the motor, as seen in Figure 7A. The dynamometer is a commercial device that does not allow real-time data collection with a data acquisition device; instead cameras were used as sensors to record the results of the dynamometer during the test. A second camera was used to record the load cell results displayed on a monitor to relate tendon force to grip force overtime. The location of the cameras is seen in Figure 7B.

The experiment starts by placing the dynamometer on the prosthesis in the grip position, as shown in Figure 7A camera 2 view. In the resting or initial position, the zero in the load cell is established. To determine the maximum grip force generated by the prosthesis, the present study measured grip force using a power grip, as this type of grip is known to produce the highest force in prosthetic hands due to its use of all five fingers in its grip (Halim et al., 2019; Fransson and WINKEL, 1991). The placement of the dynamometer was carefully determined based on the movement of the prosthetic fingers to ensure optimal force measurement.

Using ROS and the Dynamixel motor position control, the motor position is increased to close the prosthetic fingers that apply force on the dynamometer. The motor position is increased to the maximum range allowed by the motor in normal mode (180°). This last position is maintained for five seconds before a return to the initial position releases the force on the dynamometer. The motor position is controlled by a potentiometer that sends the position value directly to the setpoint of the Dynamixel motor controller. The whole procedure is recorded with audio by two cameras at 60 FPS. The test was performed six times for each prosthesis.

Since the dynamometer data were not accessible for processing with a data acquisition board, the generated force data had to be collected manually from the videos recorded by camera 2. Using the video editing software DaVinci Resolve 17 (Blackmagic Design, USA), the videos from the two cameras were synchronized, using the clip alignment function based on the audio waveform that the software provides. This process was performed for the six experiments of each prosthesis, unifying the results of each prosthesis into a single video. The results were determined by finding the maximum value reached in the dynamometer (camera 2) and averaging the five values of the tendon force observed in the monitor (camera 1). Two vectors of 6 data were obtained for each prosthesis: maximum grip force (GmF) and force applied to achieve maximum grip (TmF). This test indicates which prosthesis generates more grip force and if this value allows a prosthesis to meet ADL requirements.

### 2.3 Required and dissipated energy

This test experimentally finds the energy required to open and close each prosthesis evaluated in this study. To calculate the energy, the variables involved are the distance traveled by the tendon and the force generated in the flexion tendon of each prosthesis. The required values were measured with the S-type load cell (50 kg Lexus, China) and a camera that simultaneously recorded the monitor and the displacement of the tendon in the experiment. The same electronic setup used in Section 2.2 was employed to collect the load cell data, and the test bench in Figure 7 was likewise used. Camera 1 recorded the movement of the S-type sensor during the test, allowing the displacement required for the energy calculation to be determined.

Each of the tests in this experiment starts from an initial position of an open prosthetic hand. The hand is then closed completely using the maximum range of the Dynamixel motor (180°). When the hand is fully closed, this position is held for five seconds before the prosthesis is returned to its fully open position, and the motor is returned to its initial state (Cuellar et al., 2020; Smit et al., 2015; Cuellar et al., 2019). The experiment was recorded with camera 1 at 60 FPS. This experiment was performed six times for each prosthesis (PrHand1 and PrHand2).

It is possible to calculate the required energy (R_E) and the dissipated energy (D_E) when opening and closing the prosthesis by force generated’s integral in the flexion tendon for the displacement of the prosthesis when it is closed, as shown in the Eq. 7. The energy dissipated is calculated by subtracting the energy obtained in the backward movement of the prosthesis (from closed to open) from the energy required, as shown in the Eq. 8.

$$R_{E}=\int_{0}^{l}F\left(x\right)dx,$$ (7)

where *F*(*x*) is the force exerted on the tendons until the hand closes completely and *l* is the distance traveled by the tendons to the same point.

$$D_{E}=R_{E}-\int_{0}^{l_{1}}F_{1}\left(x\right)dx,$$ (8)

where *l*_1_ is the displacement of the tendon until the hand opens completely and *F*_1_(*x*) is the force exerted on the tendons to the same point.

The tendon force is displayed in real-time through the setup monitor. The tendon displacement was recorded utilizing the open-source video processing software Kinovea beta 0.9.4 (KINOVEA, France). With this tool, it is possible to take a fixed reference point and measure the tendon displacement based on the displacement of the S-type load cell. Obtaining a sample of tendon displacement and a sample of the force on the tendon (average of the five values seen on the monitor) was performed every *n* meters of increase in tendon displacement. The calculation of *n* is independent in each video and is calculated with Eq. 9.

$$n=\frac{TDT}{\#samples},$$ (9)

where *TDT* is the total distance traveled by the S-Type load cell, and *#samples* is the number of samples to take from video.

The *n* meter increment ensures that the same amount of data is available for each test performed in the experiment. The data obtained allow a curve of tendon force vs. tendon displacement to be plotted. From each of the six tests performed for this experiment, a vector of two variables and *n* samples [tendon force, tendon displacement] was obtained. Energy R_E and D_E were calculated using the integral function of Matlab software to determine which prosthesis requires and dissipates less energy.

### 2.4 Traction force

This experiment determines the maximum weight the prosthesis can support in its flexion state (closed hand). The maximum weight supported by the prosthesis in this test is easily converted to the traction force (TrF) using the value of gravity. The cylindrical grip is used to achieve the maximum force. This type of grip distributes the load over the 4 main fingers, making it possible to generalize the maximum force on the prosthesis.

**FIGURE 8:**
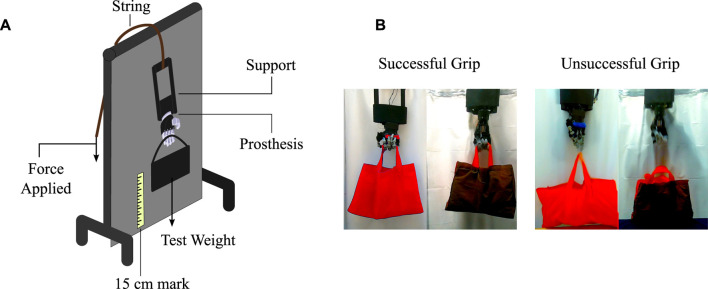
The bench needed to perform the experiment to calculate the traction force. **(A)** The components of the traction test. **(B)** Definition of successful and unsuccessful grips used in the traction test.

The primary variables involved in this experiment are the weight supported by the prosthesis (kg) and the traction force (N). The secondary variables are the length of time the weight is lifted and the distance the prosthesis has to lift the supported weight. For both prostheses, the same power supply (12V, 5A) and the same motor (Dymanixel MX-106) were used to achieve hand closure. In this experiment, sensors were not used to obtain the test data. The test bench used to find the traction force of each prosthesis is seen in Figure 8A. The setup shows the vertical location of the prosthesis, the container where the weight is placed, and how to lift the prosthesis using a string attached to a prosthesis’s base.

The test consists of placing an initial weight of 1 kg in the container held by the prosthesis, followed by the application of force on the prosthesis’s string to lift the weight of the container. The prosthesis must move 15 cm from the reference, then hold this position for 10 s before returning to the reference position (Choi et al., 2017; Mio et al., 2019). The whole procedure is performed at low speeds to avoid accelerations and abrupt forces. If the prosthesis does not suffer any mechanical damage or does not drop the weight of the container, the weight is increased in 1 kg increments and the procedure is repeated. Examples of successful and unsuccessful grips from the test are illustrated in Figure 8B. When the prosthesis fails to perform the entire test, the weight at which it fails is recorded and reduced 2 kg before performing the test again. For each test, three unsuccessful grips are obtained to allow three values for the analysis. A failure analysis is carried out after each unsuccessful grasp.

The failure report is made by quantifying the damage of each prosthesis according to four categories: permanent mechanical damage, permanent aesthetic damage, temporary mechanical damage, and temporary aesthetic damage. In the category of permanent mechanical damage, the number of broken, fractured, or bent elements are counted. The damage must make it impossible for the prosthesis to function, and the element must be replaced by a new one to repair the damage. Permanent aesthetic damage is when some of the external parts of the device break and must be changed for it to function. In this category, such damage relates to fabrics and silicone coatings. In the category of temporary mechanical damage, elements should be quantified that make it impossible for the device to function due to dislocations, sliding between tendons, or any other damage. However, the elements do not need to be exchanged for replacements; only the failed element is assembled, joined, or corrected. Finally, damage to elements such as silicone coatings and external fabrics which can be solved with adhesive are considered temporary aesthetic damage. The total number of failures will demonstrate which prosthesis suffered the most damage during the traction force test.

### 2.5 Data analysis

The statistical analysis of the three tests was carried out in two ways: (i) descriptive statistics to organize and visualize the data graphically from the mean and deviation, and (ii) inferential statistics to find the relevant differences between the two prostheses in each test. This analysis compares the mean results of the two prostheses and defines if there are significant differences between them. The inferential tests were the Mann-Whitney test and t-test according to the normality and the variance of the data. The Shapiro-Wilk test verified the normality, and the variance between the data was analyzed according to the f-test. The statistical analysis was implemented in RStudio (Version 1.3.1093,USA).

## 3 Results

This section describes the results after data processing is performed for each test. For each result set, a descriptive statistics procedure was used to visualize the results, and an inferential statistics procedure was performed to find significant differences between the PrHand1 and PrHand2 prostheses. See Supplementary Material for a video summary.

### 3.1 Grasping force

**FIGURE 9:**
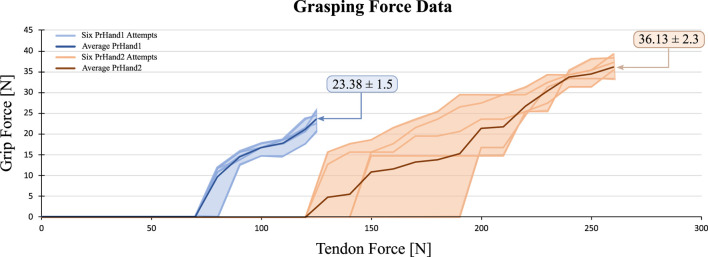
Visual representation of the grip force vs. tendon force results for 6 attempts on PrHand1 and PrHand2 prosthetic hands.

Regarding the grip force test, Figure 9 shows the grip force versus tendon force. these data were taken from the videos of camera 1 and 2. The Figure 9 shows the 6 experiments and the average of the results. It is evident that the PrHand1 prosthesis reaches the maximum GmF value much earlier than the PrHand2 prosthesis. In general, it can be seen that PrHand2 achieves a higher grip force value than PrHand1, but requires more tendon force to achieve it. The maximum values for both prostheses are: 23.38 ± 1.5 N for PrHand1 compared to PrHand2’s 36.130 ± 2.3 N of GmF. PrHand1 achieves 127.16 ± 2.8 N and PrHand1 achieves 251.81 ± 15 N of TmF.

The GmF coefficient of variation (CV) of this test is calculated. For PrHand1, a CV of 7.22% is obtained, and for PrHand2, 6.95%. This means that the variation between the results according to the mean in grip force is moderate. Therefore, it is essential to perform a statistical test to ensure significant differences between the mean values to confirm if one of the prostheses generates more grip force than the other. The data’s normality, the variance between them, and the number of results for each prosthesis were reviewed to select the inferential statistical test. For the GmF variable, the data follow a normal distribution and homogeneous variance, so a t-test is performed. This test confirmed significant differences in the results of the GmF, with a *p*-value = 1*e* ^−6^, signifying that PrHand2 generates more grasping force than PrHand1.

An inferential statistical test was likewise performed to identify a significant difference in the results of TmF in the two prostheses. The initial results for PrHand1 indicate there is a minor variation between the data (CV = 2.44%), whilst great variation (CV = 6.66%) is shown in PrHand2.

For the TmF of this test, the six results for each prosthesis follow a normal distribution. However, the variance is not homogeneous among the data, so a t-test with Welch’s correction was used. The *p*-value for the TmF in the tendon is = 5*e* ^−6^. The result indicates a significant difference between PrHand1 and PrHand2 in terms of TmF, indicating PrHand1 generates less force on the tendon than PrHand2 under the same conditions. For the statistical tests, a reliability value of 0.05 was assumed so that significant differences were present in the two variables. The summary of the grip force test results is presented in Table 2.

**TABLE 2:** Summary of mechanical test results.

	PrHand1	PrHand2	*p*-value
TmF [N]	127.16 ± 2.80	251.81 ± 15.00	5*e* ^−6^
GmF [N]	23.38 ± 1.50	36.13 ± 2.30	1*e* ^−6^
R_E [J]	0.76 ± 0.13	1.28 ± 0.13	4*e* ^−5^
D_E [J]	0.21 ± 0.17	0.96 ± 0.12	2*e* ^−6^
TrF [N]	173.31 ± 5.70	78.48 ± 0.00	0.001

### 3.2 Required and dissipated energy

**FIGURE 10:**
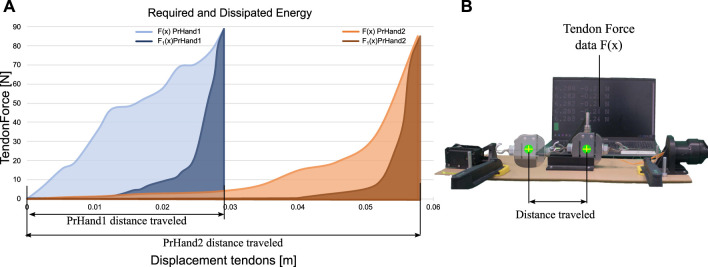
Comparison of energy required and dissipated in prosthetic devices. **(A)** Data showing energy required and dissipated during operation of prosthetic devices. **(B)** Image illustrating the calculation of the distances required for energy variables.

The results of the energy test were consolidated into two variables. The required energy R_E, as seen in Eq. 7, and the dissipated energy D_E (see Eq. 8). Regarding R_E, PrHand1 obtained a value of 0.76 ± 0.12 J and PrHand2 a value of 1.28 ± 0.13 J. The results of the D_E variable were 0.21 ± 0.17 J and 0.96 ± 0.12 J for PrHand1 and PrHand2, respectively. The results of one of the experiments in our study of energy required and dissipated are shown in Figure 10. The visual representation provided by the figure makes it easy to understand the importance of each variable in the equations used for the calculation.

In both results, there is a considerable difference between the averages of PrHand1 and PrHand2. However, the value of the CV in the results is high. The CV of R_E of PrHand1 is 15%, and for PrHand2 is 10%. The CV of PrHand1 in the D_E was 72%, and for PrHand2 was 13%.

To confirm if the difference between the averages of the results generates significant differences, the Student’s t-test was performed on unrelated samples. The data of the two prostheses follow a normal distribution, and the variance is homogeneous. These inferential tests were as follows: *p*-value of 4*e* ^−5^ for the R_E and a *p*-value of 2*e* ^−6^ for the D_E. Assuming test reliability of 5%, it can be stated that there are significant differences between PrHand1 and PrHand2 for the R_E and D_E in opening and closing the hand. The results show that the PrHand1 device requires and dissipates less energy, making it more efficient than the PrHand2 device for the same task.

### 3.3 Traction force

In this experiment, two results were obtained: a numerical result and a condition report of the prostheses after the test. The numerical result is the average maximum traction force (TrF), and the report on the condition of the prosthesis specifies the number of damaged mechanical or aesthetic elements. The numerical results for the TrF variable showed that PrHand1 measured 173.31 ± 5.7 N compared to PrHand2’s 74.48 ± 0 N.

Table 3 quantifies the permanent and temporary failures that the prostheses suffered during the test. According to the results, PrHand2 completed the traction test and underwent fewer failures than PrHand1.

**TABLE 3:** Mechanical failure report.

	PrHand1	PrHand2
Permanent mechanical damage	0	0
Permanent esthetic damage	4	0
Temporary mechanical damage	1	0
Temporary esthetic damage	0	4
Total	**5**	**4**

The result was put in bold to emphasize.

**FIGURE 11:**
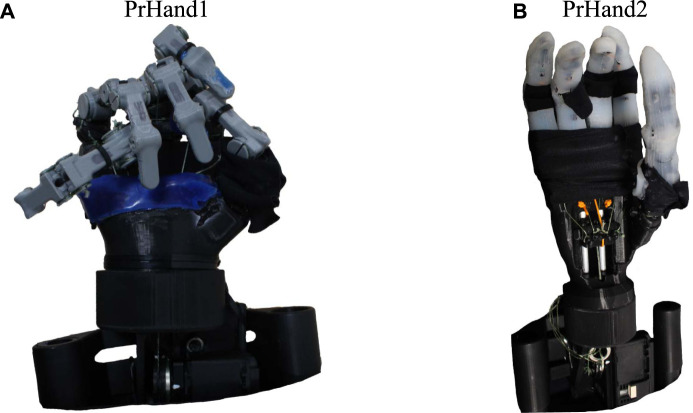
Prosthesis condition after performing the maximum supported traction test. **(A)** PrHand1 prosthesis with a dislocated finger (separated from the base). **(B)** PrHand2 prosthesis with aesthetic failures in the finger coverings.

Expanding on the values in Table 3, neither prosthesis experienced permanent mechanical damage. In the category of permanent aesthetic damage, the coverings of the four main fingers of PrHand1 wholly detached and therefore received a value of 4. The PrHand1 also suffered a dislocation of the middle finger (Figure 11A), which generated an unsuccessful grip in the test and prevented the prosthesis from functioning. This mechanical dislocation was temporary damage since the repositioning of the finger did not affect the subsequent functioning. Regarding temporary aesthetic damage, the finger coverings of PrHand2 broken and one of the joint pads detached from the finger, as seen in Figure 11B. This damage could be fixed with adhesive. PrHand1 did not experience any temporary aesthetic damage.

Unsuccessful grasps of PrHand2 always occurred at the same weight (8 Kg), and as the variation of weights in the test was performed every 1 kg, there are no intermediate values in the measurement, generating this zero standard deviation. for that reason, it is not possible to perform traditional inferential statistical tests to compare the means. However, to confirm the difference between the TrF results variable, the one-sample t-test was performed. The result of this statistical test confirms a significant difference in the TrF with a *p*-value = 0.001.

## 4 Discussion

This section presents an interpretation of the results, a discussion of how the results may be helpful in the development of prostheses, a comparison with similar studies, and limitations of the study.

### 4.1 Gripping force

The grip force test results show that PrHand2 have 54.5% stronger grip to PrHand1 since it generates greater GmF, and there is a statistically significant difference. Achieving high values in this variable is positive for a prosthesis because it allows for the manipulation more ADL objects. Mechanically, the GmF variable results can be associated with the complete silicone coating of PrHand2. This coating facilitates the hand dynamometer’s grip and reduces the loss of force due to the sliding of the sensor. The unification system is another reason PrHand2 generates more GmF: the elastic tendons of the unification system are shorter and allow less elongation, increasing energy transmission. This allows a better transfer of force from the motor to the finger flexion in the prosthesis.

The results of TmF show that 98% more force was applied in the tendon of PrHand 2 than in PrHand1. This result demonstrates that the forces opposing flexion motion in the fingers are generated by the silicone coatings and the internal path of the elastic tendon in the fingers are higher in PrHand2.

It is possible to use the GmF value to estimate if the prosthesis is functional for ADL requirements. It is estimated that values in the range of 10 N of grip strength allow for robust use of a hand prosthesis (Smit and Plettenburg, 2010b). Based on this, PrHand1 and PrHand2 can be used without grip force limitations for ADL since PrHand1 is 133.8% above the 10 N reference and PrHand2 is 261% above it.

Although the reduction in an actuator’s price, size, and weight is not a priority in the research prostheses (Tian et al., 2017b), the unification system of PrHand2 is more efficient according to the results of this test. This difference allows a smaller, more accessible motor to be used in developing countries.

It is necessary to compare the performances of PrHand1 and PrHand2 as well as results from the literature. However, the previous research does not use the same actuators or conditions as the experiment performed in this study, so the fairest way to compare the results is to select a force on the tendon as input and compare the grip force only. Grip force values generated at 100 N of applied force on the tendon were found in different studies so that the value is the point of comparison (Cuellar et al., 2020; Moreo, 2016; Cuellar et al., 2019; Smit et al., 2015; Smit, 2020). As the tendon force results of this study are not 100 N, a review of the videos that collected the data from the experiment was necessary. The review found that PrHand1 exerts 16.5 N on the grip at 100 N input, while PrHand2 did not generate a force on the dynamometer at 100 N input. PrHand1 generates similar values to the prostheses in the current literature. For example, the rigid prosthesis constructed by Cuellar et al. Cuellar et al. (2020) generated 16.84 N of grip force, which is not significantly different from the value generated by PrHand1. Two prostheses from the literature generate 15 N in the grip (Smit et al., 2015 Smit, 2020), and there is a statistically significant difference between this value and the 16.5 N of PrHand1. This difference indicates that PrHand1 generates more force than the rigid prostheses constructed by Smit et al. Smit et al. (2015); Smit (2020), as well as generating 175.23% more force than the 2019 prosthesis 2019 (Cuellar et al., 2019), which generates 6 N of grip force. The prosthesis built by Moreo et al. Moreo (2016) achieved values of almost 20 N in the grip force test with 100 N of tendon input. All statistical tests performed to compare data from the literature studies were performed with the one-sample t-test. These results show that PrHand1 generates competitive values using new technologies. The summary of the literature comparison is presented in Table 4.

**TABLE 4:** Mechanical compilation of literature prosthesis results.

	GmF [N]	R_E [J]	D_E [J]	TrF [N]
Cuellar et al. (2020)	16.84	0.38	0.32	-
Smit et al. (2015)	15.00	0.88	0.64	-
Smit (2020)	15.00	-	-	-
Moreo (2016)	20	-	-	-
Cuellar et al. (2019)	6	0.10	0.05	-
Choi et al. (2017)	-	-	-	226.22
Mio et al. (2019)	-	-	-	112.40
PrHand1	16.5	0.76	0.21	173.31
PrHand2	0	1.28	0.96	74.48

Prosthetic hand development aims to reduce the force required on tendons to increase efficiency. One way to achieve this is by optimizing the tension in the extension tendon. This can lead to a reduction in the force required in the flexion tendons, and ultimately, to achieving the lowest possible tension in the extension tendon while allowing the fingers to return to their initial position. In this particular study, the optimization process was not implemented due to the lack of instrumentation to measure pre-tension in each tendon. However, the study provides valuable insights into the potential benefits of optimizing tension in the extension tendon. It is worth noting that optimizing the tension in the extension tendon would not only reduce the total force required in the tendons, but would also increase the force of the grip. This is because the prosthesis would be able to use its resources more efficiently, resulting in a stronger grip for the user. Therefore, integrating a sensor to measure pre-tension accurately would be necessary to adjust the tension in the extension tendon, making the prosthetic hand more functional and effective for users. Specifically, the optimization process would focus on finding the pre-tension in the extension tendon that generates the highest grip force for each finger and allows the finger to return to its initial position. This could be done by iteratively adjusting the pre-tension until the desired results are achieved. Alternatively, a differential model of the tendon and force or a finite state simulation could be used to find the optimum pre-tension (Buccino et al. 2022).

### 4.2 Required and dissipated energy

The energy test results calculate which device requires the least amount of energy to operate and which dissipates the least energy, indicating the most energy-efficient device. The percentage of energy dissipated is derived from losses due to friction, heat, or deformations in the device elements. The PrHand1 prosthesis requires 39.4% less energy than the PrHand2 prosthesis, indicating significant mechanical differences in the devices. As the test was performed with the same power system (both mechanical and electrical), the variation in the results are caused by the design differences presented in Section 2, particularly the partial silicone coating of the fingers and the frictionless unification system of the PrHand1 prosthesis. These differences contribute to reducing the force of the hand closure by reducing the required energy of the system. In contrast, these same systems in the PrHand2 prosthesis make the hand closure movement more difficult. The complete covering of the fingers, the extension tendon that generates a force in opposition to the movement, and the rail-based unification system increase the required energy in this prosthesis.

PrHand1 dissipates only 27.6% of the energy it requires, while PrHand2 dissipates 75% of the required energy, which is inefficient. The energy dissipated is evidence of how easily the hand returns to its initial position, which directly links to the unification mechanism, the finger coating, and the extension tendons in each prosthesis. Although the extension tendons and silicone coating of PrHand2 help in the finger return, the friction generated in the rods of the sliding unification mechanism is high; as a result, the energy dissipation is due to the unification mechanism and the friction generated from it. An improvement for future iterations of the device should reduce the friction in the unification mechanism and reduce the opposing force of the extension tendon in the fingers.

The results of this test are relevant for commercial prostheses needing to reduce power consumption and energy waste whilst employing smaller batteries and increasing the time between recharges. Although both devices can be used in assistive applications, PrHand2 would have fewer operating hours than PrHand1 with the same battery.

PrHand2 requires 45.5% more energy than the most energy-intensive prosthesis of those reviewed in the literature (Smit et al., 2015), whereas PrHand1 is in alignment with other research. For example, there are no significant differences between the values of required energy (0.88 J) presented by Smit et al. Smit et al. (2015) and PrHand1. However, PrHand1 requires 104% and 676% more energy than the prostheses of Cuellar et al. Cuellar et al. (2020) and Cuellar and Smit et al. Cuellar et al. (2019), respectively.

Regarding dissipated energy, PrHand2 has higher values compared to the prostheses reviewed in the literature. It dissipates 50% more energy than (Smit et al. 2015). In contrast, PrHand1 only has significant differences with the prosthesis by Smit et al. (Smit et al. 2015), and the energy dissipated by PrHand1 is 67% less. There are no significant differences in the energy dissipated compared to the other prostheses reviewed (Table 4).

PrHand1 requires and dissipates energy values similar to the values of the prostheses currently considered state of the art. Compared to the literature, the novel compliant mechanism used in both prostheses requires more energy to actuate than traditional systems. The energy reduction depends mainly on the friction in the unification mechanism and the opposing force generated by the silicone coating and the extension tendons in each finger. Improvements for future iterations of the prosthesis will reduce the friction in the unification mechanism and reduce the opposing force of the extension tendon in the fingers.

### 4.3 Traction force

The final experiment identified that the PrHand1 prosthesis achieves 132.7% more traction force than the PrHand2 prosthesis. The difference is due to the energy dissipation. The cause of PrHand2’s unsuccessful grips in the traction test was due to the motor overheating and not to mechanical failures as expected. In the PrHand1 prosthesis, the unsuccessful grips were generated by dislocation of the middle finger and an overstretched tendon, indicating the prosthesis does not distribute the loads proportionally to each finger. This does not happen in the mechanism of the PrHand2 prosthesis since it allows the tensioning of each tendon and enables the adjustment of each finger if necessary. However, as the energy required to close this prosthesis is high, little power remains to lift the weight in the test, leading to the motor overheating at the same weight in each trial.

Based on the failure report in Table 3, PrHand1 finished the test with more aesthetic damage than PrHand2, but the mechanisms of PrHand2 were not pushed to the extreme due to the excessive power consumption of the motor. Of the categories evaluated in the report, the only permanent failure suffered by PrHand1 was the detachment of the silicone patch on the fingertips. As this is an easy fix, it is not considered serious damage. The finger dislocation that was the primary cause of PrHand1’s unsuccessful grip is an easily correctable failure that did not damage the device.

This test is typically destructive to rigid prostheses since high weight-bearing values cause permanent damage to axes or elements such as gears and pinions. However, using new design techniques such as novel compliant joints and elastic elements allows testing without severe damage, which demonstrates the advantages of these technologies for applications in commercial systems.

For example, the PrHand1 frictionless unifying mechanism is desirable in future iterations because this mechanism reduces the required energy needed to control the system. From the PrHand2 design, the finger coating allows a better grip and thus a higher grasping force, which is an essential element for future versions.

## 5 Conclusion and future work

Two versions of underactuated hand prostheses were constructed and mechanically evaluated to develop new technologies based on soft robotics and compliant joints. This paper presents the mechanical evaluation of the grip force, required energy/dissipated energy, and traction force. Although both versions of the PrHand prosthesis generate sufficient force for the user to perform typical ADLs, PrHand2 can generate a higher grip force with the same actuation system. The energy test shows that PrHand1 has a more efficient system because it requires less energy to close the hand, and the energy dissipation is less than 30%. In contrast, PrHand2 requires more energy and dissipates more than 70%. The energy consumption of the PrHand2 prosthesis is associated with the full silicone coating on the fingers and the sliding mechanism in the unification system. Therefore, it is necessary to redesign the unification system to improve this system. Finally, the traction force supported by PrHand1 is greater than that supported by PrHand2. This is due to PrHand2’s motor overheating and is also associated with the motor’s prosthesis consumption.

This study illustrates that both prosthesis designs built with soft robotics techniques and compliant mechanisms meet the mechanical requirements necessary to carry out ADL requirements. In addition, it suggests that these technologies are robust and efficient for this type of prosthesis application as neither design suffered a permanent failure. Based on this research, a third version is proposed to improve the performance by combining the best characteristics of each design. Our forthcoming study involving prostheses will focus on evaluating their functionality and various types of grips. To accomplish this, we will replicate the AHAP protocol (Llop-Harillo et al., 2019) and utilize 28 objects commonly used in daily living activities to assess the prostheses’ performance. Additionally, we plan to design an object capable of measuring grip force at various points along the finger to obtain more precise and real-time data for analysis. Based on the results obtained from these evaluations, we will subsequently assess a version with enhanced mechanical and functional performance with amputee patients.

## Data Availability

The original contributions presented in the study are included in the article/Supplementary Material, further inquiries can be directed to the corresponding author.
